# Robot-assisted transvesical partial cystectomy for leiomyoma of bladder trigone

**DOI:** 10.1590/S1677-5538.IBJU.2018.0801

**Published:** 2020-01-10

**Authors:** Marcos Tobias-Machado, Cristiano Linck Pazeto, Rafael Castilho Borges

**Affiliations:** 1 Faculdade de Medicina do ABC Santo AndréSP Brasil Disciplina de Urologia, Faculdade de Medicina do ABC, Santo André, SP, Brasil;; 2 Departamento de Urologia Instituto do Câncer Dr. Arnaldo Vieira de Carvalho São PauloSP Brasil Departamento de Urologia, Instituto do Câncer Dr. Arnaldo Vieira de Carvalho, São Paulo, SP, Brasil;; 3 Serviço de Urologia Hospital Alemão Oswaldo Cruz São PauloSP Brasil Serviço de Urologia, Hospital Alemão Oswaldo Cruz, São Paulo, SP, Brasil

## Abstract

**Introduction and objective:**

Leiomyomas of the urinary bladder are very rare neoplasms and are the most common benign mesenchymal tumors of the bladder, accounting for 35% of these. Treatment of leiomyomas is mainly surgical and approaches range from transurethral resection to open segmental resection or laparoscopic partial cystectomy. We sought to present the surgical technique of robot-assisted transvesical partial cystectomy for bladder leiomyoma.

**Materials and methods:**

A 25-year-old man presented to urology department with urinary frequency and urgency. Ultrasound and MRI examinations revealed a 30x20mm oval mass in the posterolateral aspect of bladder wall suggestive of bladder leiomyoma. Patient was submitted to cystoscopy with placement of a right ureteral stent and lesion demarcation, ans then a robot-assisted partial cystectomy with the following steps was performed: opening of peritoneum over bladder dome and dissection of perivesical fat, opening of bladder wall, incision of bladder mucosa, sharp and blunt dissection of lesion, closure of bladder layers with a knotless closure device.

**Results:**

Procedure was performed in 2 hours and there were no complications. Blood loss was minimal (50ml), patient was discharged after 24 hours and bladder catheter was removed after 5 days. Histopathological evaluation revealed a bladder leiomyoma with negative surgical margins.

**Conclusion:**

Robot-assisted partial cystectomy is a feasible modality for treatment of intravesical bladder leiomyomas, facilitating transvesical resection and reconstruction.

Available at: http://www.intbrazjurol.com.br/video-section/20180801_Tobias-Machado_et_al

